# Supplementary material for: High sensitivity (zeptomole) detection of BODIPY-labelled heparan sulfate (HS) disaccharides by ion-paired RP-HPLC and LIF detection enables analysis of HS from mosquito midguts
Source: Anal Methods. 2023 Feb 2;15(11):1461–9. doi: 10.1039/d2ay01803a (PMC10019443; doi:10.1039/d2ay01803a)
Supplement: AY-015-D2AY01803A-s001 [file AY-015-D2AY01803A-s001.pdf]

Electronic Supplementary Material (ESI) for Analytical Methods.
This journal is © The Royal Society of Chemistry 2023

High sensitivity (zeptomole) detection of BODIPY- labelled heparan sulfate (HS) disaccharides by ion-paired RP-HPLC and LIF detection enables analysis of HS from mosquito midguts.

Marissa L. Maciej-Hulme[1], Anaëlle C. N. Leprince[1,2], Andre Lavin[3], Scott E. Guimond[1], Jeremy E. Turnbull[1], Julien Pelletier[1], Edwin A. Yates[4,1], Andrew K. Powell[3] and Mark A. Skidmore[1,4]

1. Centre for Glycoscience Research and Training, School of Life Sciences, Keele University, Staffordshire, ST5 5BG, United Kingdom.
2. Université de Rennes 1, Rue du Thabor, 35065 Rennes Cedex, France.
3. School of Pharmacy and Biomolecular Sciences, Liverpool John Moores University, Liverpool L3 3AF, United Kingdom.
4. Department of Biochemistry and Systems Biology, Institute of Systems, Molecular and Integrative Biology, University of Liverpool, Liverpool L69 7ZB United Kingdom.

SUPPLEMENTARY INFORMATION

Supplementary Figures

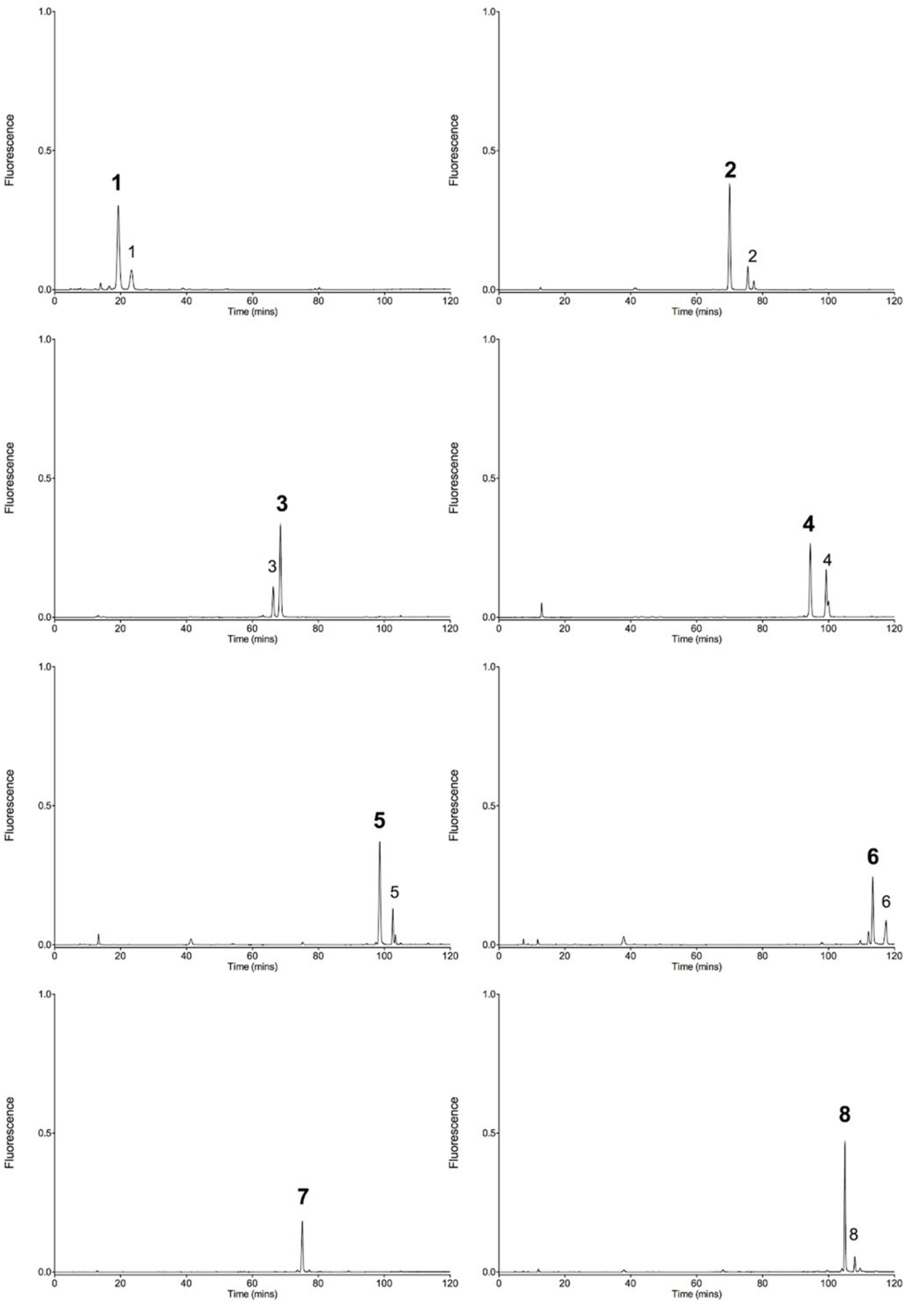

Figure S1. Chromatograms of BODIPY-labelled individual HS Δ-disaccharides 1 - 8. Dominant peaks are noted in bold, minor peak species are also labelled.

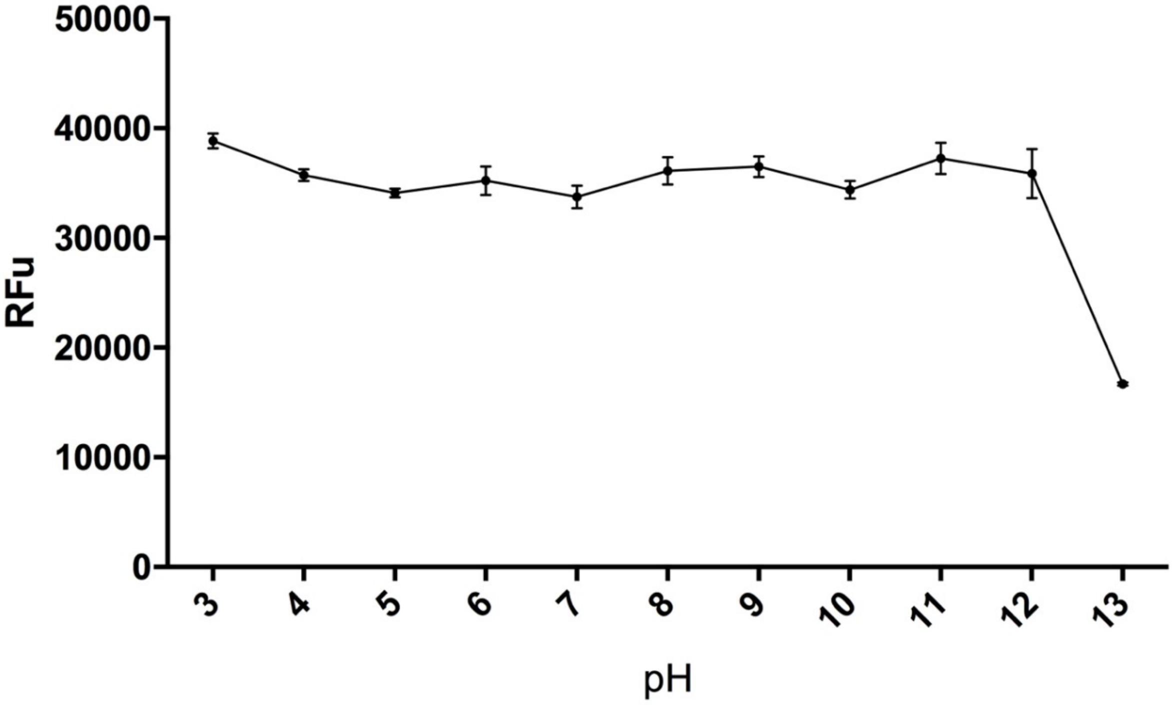

Figure S2. BODIPY-FL hydrazide fluorescence in water pH 3 - 13. Error bars represent the SEM of three replicates.
